# Supplementary material for: Breaking the crosstalk of the Cellular Tumorigenic Network by low-dose combination therapy in lung cancer patient-derived xenografts
Source: Commun Biol. 2022 Jan 17;5:59. doi: 10.1038/s42003-022-03016-5 (PMC8763947; doi:10.1038/s42003-022-03016-5)
Supplement: Supplementary file 2 — Supplementary Information [file 42003_2022_3016_MOESM2_ESM.pdf]

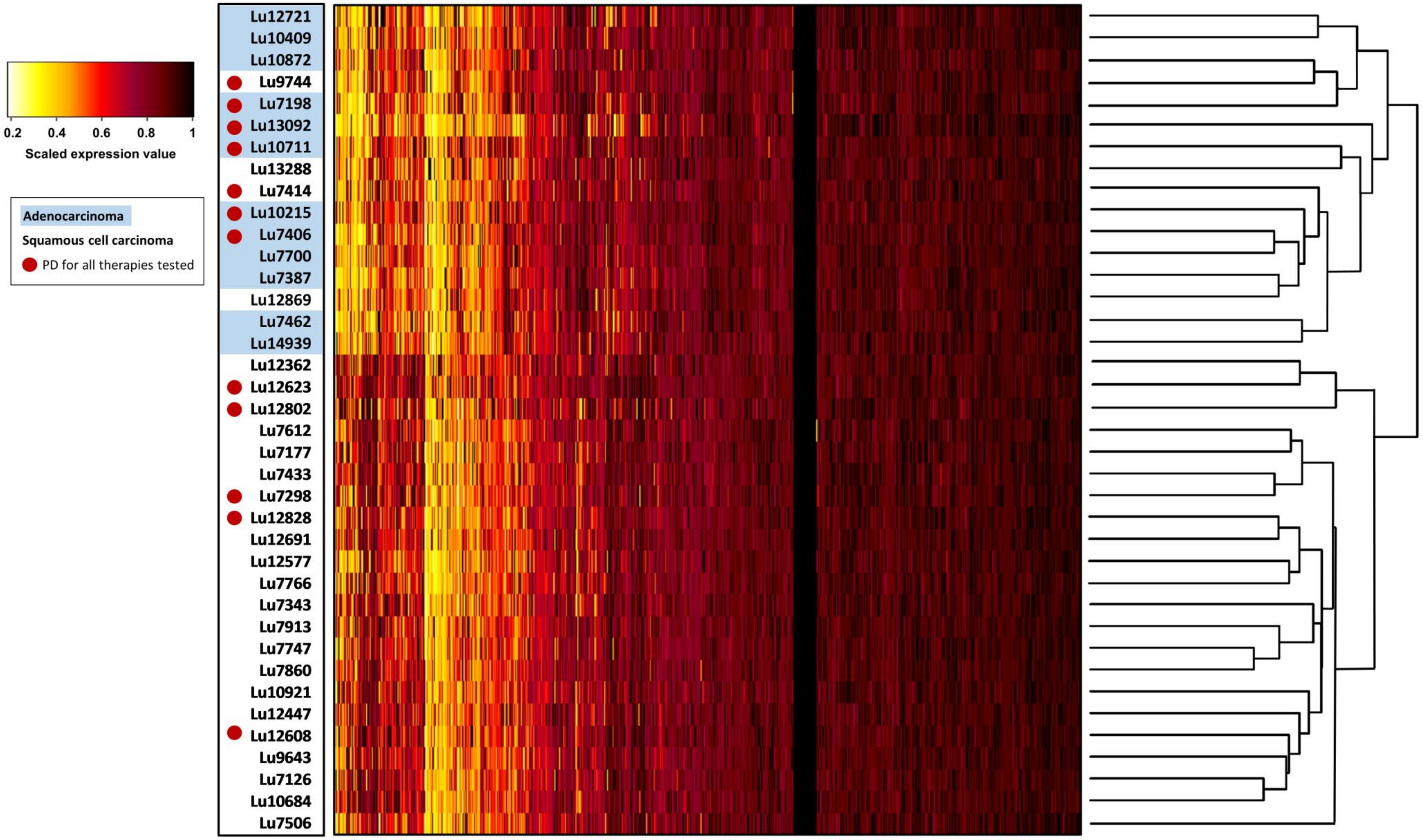


**Supplementary Figure 1**

The heatmap represents the relative expression of 19096 HGNC-annotated genes in 38 NSCLC tumors from the EPO tumor biobank. NSCLC tumors with progressive disease (PD) as best response evaluated in patient-derived xenografts are indicated and highlighted with a red circle. Hierarchical clustering of the expression data results in two main clusters reflecting the histology-based gene expression of adenocarcinoma and squamous cell carcinoma PDX. Raw data are available from the corresponding author upon request.
