# Supplementary material for: Breaking the crosstalk of the Cellular Tumorigenic Network by low-dose combination therapy in lung cancer patient-derived xenografts
Source: Commun Biol. 2022 Jan 17;5:59. doi: 10.1038/s42003-022-03016-5 (PMC8763947; doi:10.1038/s42003-022-03016-5)
Supplement: Supplementary file 3 — Description of Additional Supplementary Files [file 42003_2022_3016_MOESM3_ESM.pdf]

Description of Additional Supplementary Files

File name: Supplementary Data 1
Description: Source data for the graphs and charts in the main figures.
