# Supplementary material for: Breaking the crosstalk of the Cellular Tumorigenic Network by low-dose combination therapy in lung cancer patient-derived xenografts
Source: Commun Biol. 2022 Jan 17;5:59. doi: 10.1038/s42003-022-03016-5 (PMC8763947; doi:10.1038/s42003-022-03016-5)
Supplement: Supplementary file 5 — Reporting Summary [file 42003_2022_3016_MOESM5_ESM.pdf]

nature portfolio

Corresponding author(s): Stefan Langhammer

Last updated by author(s): Dec 23, 2021

Reporting Summary

Nature Portfolio wishes to improve the reproducibility of the work that we publish. This form provides structure for consistency and transparency in reporting. For further information on Nature Portfolio policies, see our Editorial Policies and the Editorial Policy Checklist.

Statistics

For all statistical analyses, confirm that the following items are present in the figure legend, table legend, main text, or Methods section.

n/a	Confirmed	
☐	☒	The exact sample size (*n*) for each experimental group/condition, given as a discrete number and unit of measurement
☐	☒	A statement on whether measurements were taken from distinct samples or whether the same sample was measured repeatedly
☐	☒	The statistical test(s) used AND whether they are one- or two-sided<br>*Only common tests should be described solely by name; describe more complex techniques in the Methods section.*
☒	☐	A description of all covariates tested
☐	☒	A description of any assumptions or corrections, such as tests of normality and adjustment for multiple comparisons
☐	☒	A full description of the statistical parameters including central tendency (e.g. means) or other basic estimates (e.g. regression coefficient) AND variation (e.g. standard deviation) or associated estimates of uncertainty (e.g. confidence intervals)
☐	☒	For null hypothesis testing, the test statistic (e.g. F, t, r) with confidence intervals, effect sizes, degrees of freedom and P value noted<br>*Give P values as exact values whenever suitable.*
☒	☐	For Bayesian analysis, information on the choice of priors and Markov chain Monte Carlo settings
☒	☐	For hierarchical and complex designs, identification of the appropriate level for tests and full reporting of outcomes
☒	☐	Estimates of effect sizes (e.g. Cohen's *d*, Pearson's *r*), indicating how they were calculated

Our web collection on statistics for biologists contains articles on many of the points above.

Software and code

Policy information about availability of computer code

Data collection	No software was used.
Data analysis	For processing NGS (RNASeq) data only the following open source software tools were used: FastQC (version 0.11.8), Xenome (version 1.0.1), STAR aligner (version 2.6.1a), QualiMap (version 2.2.1) and eXpress (version 1.5.1).<br>The expression data analyses were performed in the free software environment R (version 4.0.3) for statistical computing and graphics using following packages provided by Bioconductor (version 3.7): DESeq2 (version 1.30.1), GSVA (version 1.38.2), gplots (version 3.1.1) and RColorBrewer (version 1.1-2).

For manuscripts utilizing custom algorithms or software that are central to the research but not yet described in published literature, software must be made available to editors and reviewers. We strongly encourage code deposition in a community repository (e.g. GitHub). See the Nature Portfolio guidelines for submitting code & software for further information.

Data

Policy information about availability of data

All manuscripts must include a data availability statement. This statement should provide the following information, where applicable:

- Accession codes, unique identifiers, or web links for publicly available datasets
- A description of any restrictions on data availability
- For clinical datasets or third party data, please ensure that the statement adheres to our policy

Source data for the graphs and charts in the main figures are provided in Supplementary Data 1. DNA sequencing data of the xenograft models are available at http://www.epo-berlin.com/epo-tumor-models-xenografts.html. Access will be granted by sending an email to service@epo-berlin.com.

Field-specific reporting

Please select the one below that is the best fit for your research. If you are not sure, read the appropriate sections before making your selection.

☒ Life sciences ☐ Behavioural & social sciences ☐ Ecological, evolutionary & environmental sciences

For a reference copy of the document with all sections, see nature.com/documents/nr-reporting-summary-flat.pdf

Life sciences study design

All studies must disclose on these points even when the disclosure is negative.

Sample size	The animal number of each experiment in the treatment combination group is visible in Figure 4 in which all individual animals were shown. Control groups were always conducted with n=3 animals.<br>The first experiment with LU7406 was conducted as single mouse cancer trial in the treatment combination group. Combination therapy treatment of Lu7414 was conducted in n=5 animals in total, of which three animals are displayed in Fig.3a and two animals are displayed in Fig. 3b. as indicated in the figure legends.
Data exclusions	No data was excluded from analysis
Replication	All measures were taken using calibrated instruments (digital caliper, accuracy balance) ensuring reproducibility
Randomization	Animals were randomly assigned to study groups. Tumor volume values sorted from high to low were overlayed with a pre-installed randomization scheme which was individually constructed for each experiment.
Blinding	n/a

Reporting for specific materials, systems and methods

We require information from authors about some types of materials, experimental systems and methods used in many studies. Here, indicate whether each material, system or method listed is relevant to your study. If you are not sure if a list item applies to your research, read the appropriate section before selecting a response.

Materials & experimental systems

n/a	Involved in the study	
☐	☒	Antibodies
☒	☐	Eukaryotic cell lines
☒	☐	Palaeontology and archaeology
☐	☒	Animals and other organisms
☒	☐	Human research participants
☒	☐	Clinical data
☒	☐	Dual use research of concern

Methods

n/a	Involved in the study	
☒	☐	ChIP-seq
☒	☐	Flow cytometry
☒	☐	MRI-based neuroimaging

Antibodies

Antibodies used	rabbit monoclonal anti-PD-L1 antibody (1:100, clone E1L3N, Cell Signaling #13684, Frankfurt a.M., Germany), mouse anti-human pan-Cytokeratin (1:50; clone AE1/AE3; Dako #IS05330-2, Hamburg, Germany), rat anti-mouse CD31 (1:200; clone SZ31, Dianova # DIA-310-BA-2, Hamburg, Germany), mouse anti-mouse alpha–SMA (1:400; clone 1A4; Sigma-Aldrich Chemie Taufkirchen, Germany), goat-anti-rat-HRP antibody (Dianova, Hamburg, Germany)
Validation	#13684 valid for WB, IP, IHC, IF, F, ChIP, #IS05330-2 valid for IHC, #DIA-310-BA-2 valid for WB and IHC

Animals and other organisms

Policy information about studies involving animals; ARRIVE guidelines recommended for reporting animal research

Laboratory animals	Mus musculus, Rj:NMRI-Foxn1nu/nu female mice, 6-8 weeks upon PDX fragment transplantation
Wild animals	The study did not involve wild animals
Field-collected samples	n/a
Ethics oversight	All experiments were in agreement with the Guidelines for the Welfare and Use of Animals in Cancer Research and the German

Ethics oversight	Animal Protection Law and approved by local authorities: Landesamt für Gesundheit und Soziales Berlin (LAGeSo Berlin, Germany) with the registration number A0010/19

Note that full information on the approval of the study protocol must also be provided in the manuscript.
